# Case Report: Traumatic Tension Pneumothorax in a Pediatric Patient

**DOI:** 10.21980/J8ZD1S

**Published:** 2021-01-15

**Authors:** Zachary Tritsch, Gayle Galan, Gary Oates, Janelle Thomas

**Affiliations:** * Marietta Memorial Hospital, Department of Emergency Medicine, Marietta, OH

## Abstract

This case demonstrates the importance of quickly identifying a pediatric patient in acute respiratory distress secondary to pneumothorax and highlights the emergent management of these patients. The 16-year-old male patient presented to the emergency department (ED) with a chief complaint of right-sided rib pain and shortness of breath that began acutely after an all-terrain vehicle (ATV) accident. The patient was in respiratory distress upon presentation and had diminished right-sided breath sounds. A portable chest X-ray demonstrated pneumothorax with significant mediastinal leftward shift. Needle decompression of the right chest wall was performed and right sided thoracostomy was placed. It is important to maintain a high index of suspicion for tension pneumothorax in pediatric trauma patients with respiratory distress, even when hemodynamically stable. This case report discusses proper management of traumatic tension pneumothorax and discusses current recommendations for needle decompression and thoracostomy.

**Topics:**

Tension pneumothorax, pediatrics, respiratory distress, portable chest x-ray, ultrasound.

**Figure:**
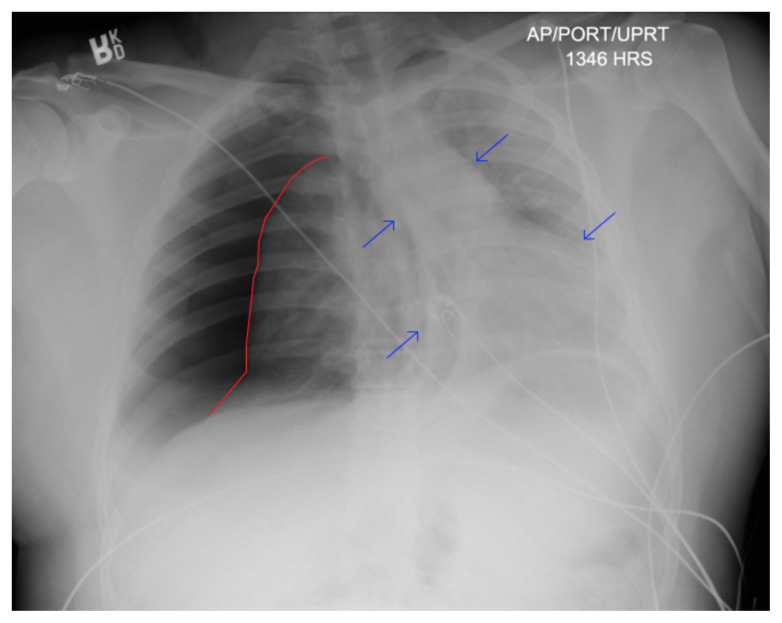

## Brief introduction

Pneumothorax occurs when free air enters the space between the visceral and parietal pleura. Tension pneumothorax can occur as a consequence of trauma and is considered one of the most dangerous types of pneumothoraces. With a tension pneumothorax, air cannot escape secondary to a one-way valve system. As a result, intrathoracic pressure increases and allows for compression of the aorta, inferior vena cava, and heart. Because the intrathoracic pressure increases, mediastinal structures are shifted laterally. This causes reduction in cardiac output, decreased diastolic filling, decreased venous return and can ultimately lead to hypotension and hypoxia. In severe cases, cardiac arrest can result from the obstructive shock and hypoxia caused by tension pneumothoraces.^1^, ^2^, ^3^ This case is unique in that it presents a tension pneumothorax in a pediatric patient. Pediatric patients are a particularly vulnerable population. Children have an increased physiologic reserve that helps to maintain systolic blood pressure in the normal range, even in shock. Physicians must be able to recognize signs of early shock such as tachycardia and poor skin perfusion because rapid clinical deterioration can ensue. Unstable pediatric patients must be treated emergently with needle decompression and closed chest thoracostomy.^2^,^4^

## Presenting concerns and clinical findings

A 16-year-old male with no significant past medical or surgical history presented to the ED after ATV accident, complaining of right sided rib pain and shortness of breath. He was unhelmeted, lost control while driving the ATV, and struck a ditch and a tree. He was traveling at an unknown speed when his vehicle crashed. He struck his chest against the steering wheel and he denies loss of consciousness. On review of systems, he had no pain of his extremities and reported right-sided abdominal pain. He was in significant distress when speaking, diaphoretic, alert and oriented, tachycardic, tachypneic, hypoxic (93% on room air), and normotensive. Breath sounds were significantly diminished on the right, and patient had 2+ pulses throughout. His abdomen was tender to palpation in right upper quadrant, and extremities were atraumatic. He had a complex laceration to the left posterior auricle, and there was a 2-centimeter scalp laceration. Stat portable chest x-ray was obtained because there was concern for pneumothorax versus hemothorax.

## Significant findings

Chest X-ray demonstrated significant right-sided pneumothorax (with red outline showing border of collapsed right lung) with cardio mediastinal shift to the left (shown by blue arrows) indicative of a tension pneumothorax

## Patient course

After stat portable chest x-ray was obtained, needle decompression was performed with a 14-gauge needle in the right second intercostal space at the midclavicular line (ICS-MCL). In preparation of chest thoracostomy, 50 mcg fentanyl was administered for pain. Initially, chest thoracostomy was attempted with a 28-gauge chest tube but was unsuccessful secondary to the patient’s narrow rib space at the right fourth ICS at the mid axillary line (MAL). The patient was administered an additional 50 mcg of fentanyl and a 24-gauge chest tube was successfully placed at right fourth ICS-MAL, and the patient’s hypoxia and tachycardia improved. Repeat chest X-ray showed re-expansion of right lung. Computed tomography (CT) imaging of chest, abdomen, and pelvis revealed extensive alveolar infiltrates and areas of atelectasis in the right upper lobe, right lower lobe, and left upper lobe. Subcutaneous emphysema was noted bilaterally and a tiny apical left pneumothorax was noted. There were no rib fractures visualized. Computed tomography imaging of head, carotid arteries, cervical, thoracic, and lumbar spine were also obtained and showed no acute bone injuries or abnormalities. Labs, including complete blood count, comprehensive metabolic panel, urinalysis, and urine drug screen, were otherwise unremarkable. Patient was transferred to an accepting children’s hospital.

## Discussion

Although traumatic thoracic injuries are uncommon in children (accounting for approximately 5% to 12% of pediatric trauma admissions), thoracic injuries in pediatric patients are the second leading cause of death from trauma after closed head injuries. Isolated chest trauma carries a 4% to 12% mortality rate; this rate increases to 40% when associated with closed head injuries and abdominal trauma. Most pediatric thoracic injuries (80% to 85%) are the result of blunt trauma, with falls, sports-related injuries, and high-speed crashes accounting for most blunt traumatic injuries in older children and teenagers.^2^

Thoracic trauma is one of the leading causes of tension pneumothorax. Chest X-rays can be obtained if diagnosis of pneumothorax cannot be made clinically and the patient is relatively stable.^2^ Although bedside ultrasound was not used in this case, ultrasound can be used to detect pneumothoraces and has been purported to have better sensitivity and specificity for detecting pneumothorax in comparison to chest X-ray; however, no imaging modality should delay treatment of a patient with an unstable tension pneumothorax.^5^ Arguably, the patient in this case who presented as tachycardic, tachypneic, and hypoxic, may have been better managed if imaging had been forgone, and the diagnosis of tension pneumothorax was made clinically. As the patient was normotensive in this case, portable chest X-ray was obtained, allowing the clinicians to make a definitive diagnosis of tension pneumothorax.

Rib fractures are rare in children secondary to children’s rib cages having increased elasticity, in comparison to adults. The patient in this case had no rib fractures present. When rib fractures are present, they are associated with a high-energy mechanism.^2^

Needle decompression can be executed at the fourth intercostal space in the anterior axillary line (ICS-AAL) or at the ICS-MCL as in the case report. Recent literature has suggested that decompression at the fourth ICS-AAL may be more favorable and this is currently recommended in current Advanced Trauma Life Support (ATLS) guidelines.^4^,^6^ Needle decompression at this area may be more favorable in pediatric patients, given that the heart and thymus can be close in proximity to the area of decompression at the second ICS-MCL.^7^ Additionally, ultrasound has been recommended to both measure chest wall thickness and look at underlying structures prior to needle decompression if time permits.^8^

There is minimal research on insertion depth of a needle for decompression. ATLS guidelines currently recommend insertion of a 5 cm, 14-gauge needle for decompression of a tension pneumothorax.^4^,^6^,^9^ In a recent study, researchers utilized CT imaging to examine appropriate needle length for pneumothorax decompression in pediatric patients less than 13 years. The study concluded that the standard 5 cm needle is twice the chest wall thickness of most children, and 14-gauge or 16-gauge standard-length 3.8 cm needles are of adequate length to access the pleural cavity in pediatric patients less than 13 years old^9^.

This case demonstrates the importance of quickly identifying pediatric patients in acute respiratory distress secondary to tension pneumothorax and emphasizes the emergent and necessary treatment these patients require. Thoracic trauma from the ATV accident was the definitive cause of pneumothorax in this case. Patients with tension pneumothorax can be unstable on presentation, and this case proves how the importance of a thorough physical exam can lead to a definitive diagnosis of tension pneumothorax. Additionally, this case demonstrates how bedside maneuvers such as a bedside lung ultrasound could have benefitted this patient. The case discusses the current recommendations regarding recommended anatomic placement of needle and size of needle for decompression tension pneumothorax.

## Supplementary Information






