# Supplementary figures and images for: Case Report: Traumatic Tension Pneumothorax in a Pediatric Patient
Source: J Educ Teach Emerg Med. 2021 Jan 15;6(1):V26–8. doi: 10.21980/J8ZD1S (PMC10332764; doi:10.21980/J8ZD1S)

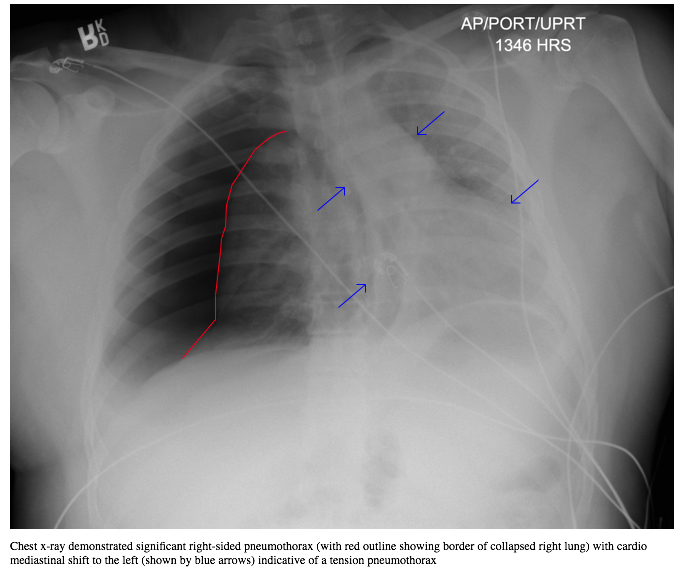

Supplement: Supplementary file 1 [file jetem-6-1-v26-supp1.jpg]

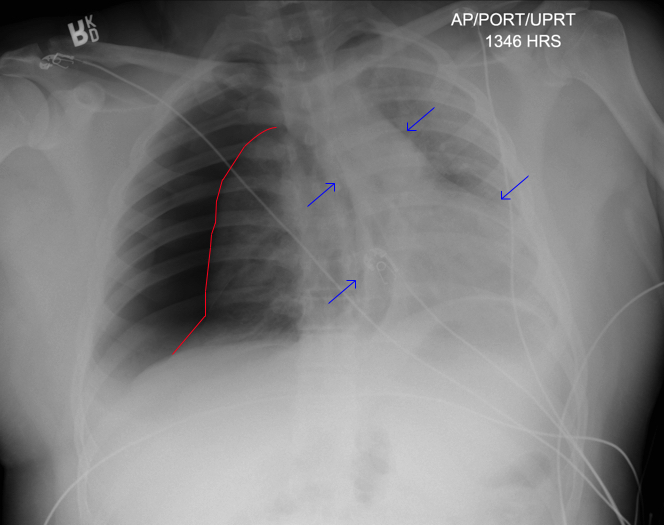

Supplement: Supplementary file 2 [file jetem-6-1-v26-supp2.jpg]

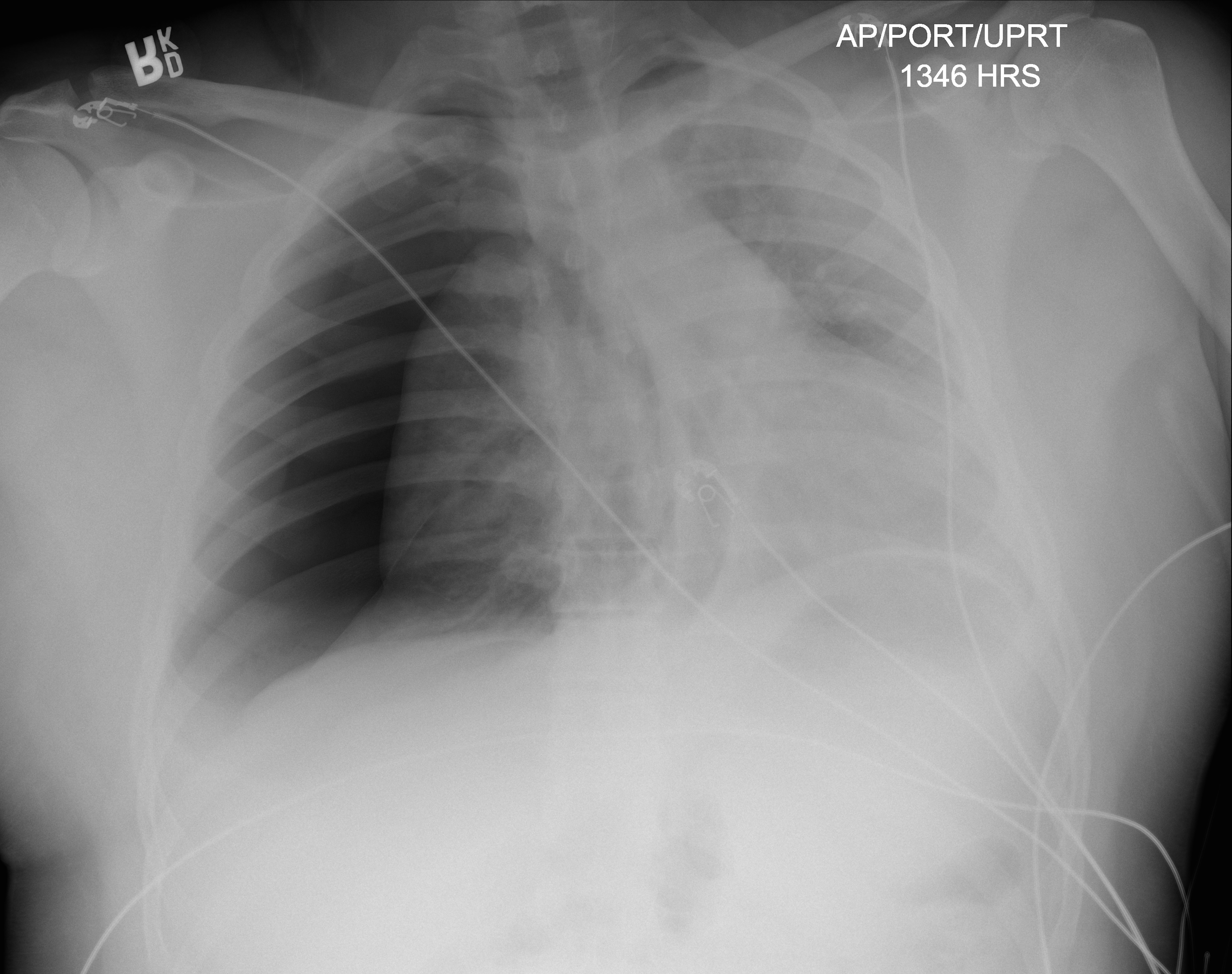

Supplement: Supplementary file 3 [file jetem-6-1-v26-supp3.jpg]
